# Automatic segmentation of deep intracerebral electrodes in computed tomography scans

**DOI:** 10.1186/s12859-015-0511-6

**Published:** 2015-03-25

**Authors:** Gabriele Arnulfo, Massimo Narizzano, Francesco Cardinale, Marco Massimo Fato, Jaakko Matias Palva

**Affiliations:** 1 Department of Informatics, Bioengineering, Robotics and System Engineering – DIBRIS, University of Genoa, Viale Causa 13, Genoa, Italy; 2 C. Munari Centre for Epilepsy Surgery, Niguarda Hospital, Piazza Ospedale Maggiore 3, Milano, Italy; 3 Neuroscience Center, University of Helsinki, P.O. Box 56 (Viikinkaari 4) FI-00014, Helsinki, Finland

**Keywords:** SEEG, Automatic segmentation, Epilepsy

## Abstract

**Background:**

Invasive monitoring of brain activity by means of intracerebral electrodes is widely practiced to improve pre-surgical seizure onset zone localization in patients with medically refractory seizures. Stereo-Electroencephalography (SEEG) is mainly used to localize the epileptogenic zone and a precise knowledge of the location of the electrodes is expected to facilitate the recordings interpretation and the planning of resective surgery. However, the localization of intracerebral electrodes on post-implant acquisitions is usually time-consuming (*i.e.*, manual segmentation), it requires advanced 3D visualization tools, and it needs the supervision of trained medical doctors in order to minimize the errors. In this paper we propose an automated segmentation algorithm specifically designed to segment SEEG contacts from a thresholded post-implant Cone-Beam CT volume (0.4 mm, 0.4 mm, 0.8 mm). The algorithm relies on the planned position of target and entry points for each electrode as a first estimation of electrode axis. We implemented the proposed algorithm into DEETO, an open source C++ prototype based on ITK library.

**Results:**

We tested our implementation on a cohort of 28 subjects in total. The experimental analysis, carried out over a subset of 12 subjects (35 multilead electrodes; 200 contacts) manually segmented by experts, show that the algorithm: (i) is faster than manual segmentation (*i.e.*, less than 1s/subject versus a few hours) (ii) is reliable, with an error of 0.5 mm ± 0.06 mm, and (iii) it accurately maps SEEG implants to their anatomical regions improving the interpretability of electrophysiological traces for both clinical and research studies. Moreover, using the 28-subject cohort we show here that the algorithm is also robust (error < 0.005 mm) against deep-brain displacements (< 12 mm) of the implanted electrode shaft from those planned before surgery.

**Conclusions:**

Our method represents, to the best of our knowledge, the first automatic algorithm for the segmentation of SEEG electrodes. The method can be used to accurately identify the neuroanatomical loci of SEEG electrode contacts by a non-expert in a fast and reliable manner.

## Background

Invasive monitoring of brain activity with intracranial ElectroEncephaloGraphy (iEEG) is widely practiced to improve diagnosis of several neurological diseases. In recent years, iEEG analyses have been also widely adopted in several research studies [1] mainly aimed at characterizing neuronal correlates of language [2], motor [3,4] as well as cognitive tasks [5]. Epilepsy is the main field of interest of diagnostic iEEG.

The American Academy of Neurology recommended that “patients with disabling complex partial (focal) seizures, with or without secondarily generalized seizures, who have failed appropriate trials of first-line antiepileptic drugs should be considered for referral to an epilepsy surgery center” [6]. The key point of successful epilepsy surgery is the correct identification of the Epileptogenic Zone (EZ), definable as “the site of the beginning and of the primary organization of the epileptic seizures” [7]. In 25% to 50% of subjects, the EZ is identified by means of iEEG recording [8-12]. The largely adopted ElectroCorticoGraphy (ECoG) is performed implanting subdural grids or strips of disk-shaped electrodes laying on the brain surface. Such electrodes do not allow direct recording from the depth of the sulci or from the white matter, and also the sampling of the mesial and inferior aspects of the hemispheres is challenging. Another common invasive approach is to implant a small number of depth electrodes (DE), mainly aimed at lateralizing temporal lobe seizures. The limited number of such DE does not provide a sufficient sampling of brain structures for the complex tri-dimensional definition of the EZ [13]. A definitely higher number of thinner and less traumatic intracerebral electrodes is used for performing StereoElectroEncephaloGraphy (SEEG), a methodology developed by Talairach and Bancaud at Hôpital Sainte Anne, Paris [14]. “Stereo-EEG with intracerebral depth electrodes is increasingly used to define the epileptogenic zone in complex cases, with a lower rate of complications than subdural grids” [15].

An accurate localization of the electrode contacts relative to cortical and sub-cortical structures allows mapping the local field potentials (LFP) to the brain improving the interpretability of the pathological networks. During clinical practice, the assessment of implant accuracy is commonly performed by visual inspection of Magnetic Resonance Imaging (MRI) and Computed Tomography (CT) scans, or intraoperative photography [16] in SEEG and ECoG, respectively.

In post-operative MR images, the electrode artifacts obscure both the exact position of each recording contact and its proximal anatomy. These artifacts result in distorted cortical sheets and contacts larger than expected indirectly compromising the contact localization. CT images allow for a more precise contact visualization in the volume since are less affected by metal artifacts. CT scans, however, have a poor soft-tissue contrast, which blurs the anatomical boundaries of cerebral structures. Thus, pre-operative MRI and a post-implant CT data are usually fused to guide neurosurgeons in the assessment of contact position and in the identification of neighbouring brain structures [17].

**Figure 1:**
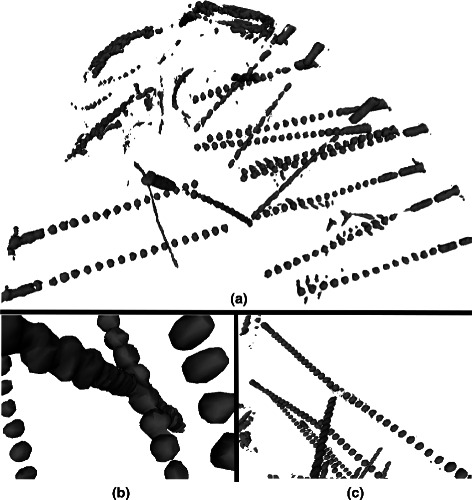
**Examples of a CT scan of a (tipical) SEEG implant, characterized by a high number of electrodes (targetting deep brain structures from the cortical surfaces).** **(a)** Gray-scale surface models representing an example of image resulting from a CT scan opportunely thresholded to separate between contact and brain tissue. **(b)** SEEG implants aim to characterize the EZ thus it is quite common that several electrodes have narrow trajectories pointing at the same region from different sides. The artifact around each contact that blurs the exact geometry of the cylinder results in electrodes that apparently touch each other or **(c)** even apparently seem to be sequential traversing the whole brain.

Electrode shafts remain straight in the cortical tissue only rarely and essential never fully lay on radiological planes (*i.e.*, axial, coronal, and sagittal) (Figure 1(a)). Despite the large amount of time needed to localize several tens to thousands points in a single volumetric image, the navigation of brain volumes along non-conventional planes strongly affects the chance to accurately discern between brain structures even for trained and expert medical doctors. Thus, image fusion of pre- and post-implant imaging datasets, despite being a useful tool, fails to help the clinical practices by not sensibly reducing the amount of manual work to neurophysiologists and neurosurgeons [18].

Moreover, due to the intrinsic aim of the methods (i.e., investigation of limited brain volume), usually SEEG electrodes have narrow and even convergent trajectories (Figure 1(b)) which sensibly limit the applicability of classical region growing approaches and of more simple threshold-based approaches.

Recently, research groups proposed several techniques for the localization of iEEG electrodes. Most of the approaches aim to simplify the manual extraction of contact coordinates by fusing opportunely thresholded post-implant datasets on pre-implant MRIs [19-21].

Other approaches take advantage of geometrical constraints (i.e., contact/electrode geometry) and seed point initialization that works on iEEG [22], subdural [23] or Deep Brain Stimulation (DBS) [24] electrodes. Even though Dykstra and colleagues approach [22] deal with deep brain electrodes, the extraction of contact coordinates is performed by visual inspection of single subject post-implant CT scans. Thus their method of implant mapping to subject space is limited to a small number of subjects at the same time due to the time requested for the visual extraction. Subdural electrodes are arranged in grid/strips which do not penetrate sulci and remain confined to the cortical surface. Taken together this aspects, automated or semi-automated segmentation methods in ECoG implants cannot be directly applied to DE. Not to mention that brain shifts in ECoG are much more significant than in SEEG case which require then a post-processing steps to account for that. The more similar scenario in terms of electrode geometry (*i.e.*, linear shafts, cylindrical multi lead-electrode, converging trajectories) is DBS used in treatment Parkinson’ Disease. Here, the limited number of contacts (*i.e.*, 4 channels/electrode) and electrodes (*i.e.*, at most one per hemisphere in case of non-lateralized disease) make the task of automatic segmentation of contacts within patients reference space easier to be solved. The proposed approach by Hebb et al. [24], takes advantage of the Hounsfield scale to discriminate between contact and tissue voxels in order to get rid of the manual selection of threshold. This approach cannot be used in our specific case for two reasons. The former is that Hounsfield scale is not reliable for Cone Beam CT scans [25]. The latter is that the larger number of electrodes/channels might result in crossing trajectories, making the task to automatically assessing at each contact the correct label unreliable.

Thus, none of these approaches is suitable for fully automatic localization of SEEG electrode contacts in their commonly complex implantation arrangements. In this paper we present a novel algorithm specifically designed to automatically segment SEEG contacts from a thresholded post-implant Cone-Beam Computed Tomography (CBCT) volume (anisotropic voxel: 0.4 mm, 0.4 mm, 0.8 mm). The accuracy and reliability of the proposed algorithm are evaluated analyzing data from 28 subjects corresponding to 439 electrodes and 5937 contacts.

## Results and discussion

In the present study, we used MRI and CT data from 28 patients undergoing pre surgical evaluation for resective surgery aimed at treating drug-resistant epilepsy. Each dataset is composed of one thresholded, skull-stripped, post-implant CT volume previously coregistered to the surgical reference space (*i.e.*, MRI); and a fiducial file where planned entry (*i.e.*, on the cortical surface) and target (*i.e.*, deepinside the brain) points are listed for each electrode. These data are mandatory to correctly associate electrode and contact labels to the relative electrophysiologic signals and as initial estimate of electrode axes.

Briefly, the algorithm searches for the best candidate of each contact traveling from entry to target points along the estimated electrode axis in order to localize the first contact located on the tip of each electrode. The above process is replicated in the opposed direction, target-entry along the same axis, to finely compute contact centroids.

**Figure 2:**
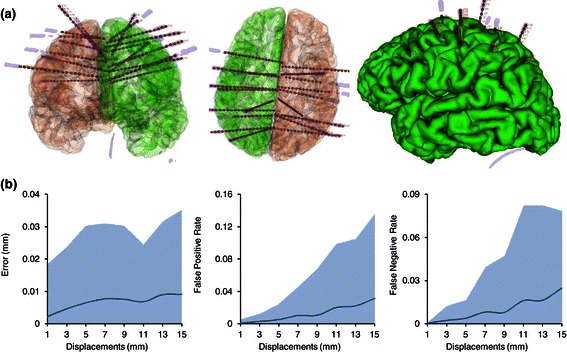
**Results of the DEETO algorithm on complex scenarios.** **(a)** The figure shows the accuracy of the reconstructed implant. The complexity of SEEG implants is well represented in the figure where it can be easily seen the high number of electrodes used, their direction, and their proximity to each others. Moreover, we superimposed pial surfaces (green - left and red - right) extracted from the MRI previously coregistered to CT space. Original electrode surface models extracted from patient CT (shaded gray) are overlayed by segmented contacts (red cylinder) with their real dimensions (*i.e.*, each cylinder is 2 mm long, 0.8 mm diameter and inter-contact distance is 1.5 mm). **(b)** The proposed implementation is robust to target point displacements. Target displacements (left plot) up to 12 mm from planned site result in an average error of 0.005 mm (blue line). The number of False Positive (FP; central plot) and False Negative (FN; right plot) divided by the total number of real contacts increase with target point displacements but remained below 10% for distances up to 13 mm. Shaded area in all plots indicate the interval between the 95th and the 5th percentiles extracted from 28 subjects and 5 samples for each distance (see methods for details).

Our method, implemented in a C++ prototype called DEETO, successfully reconstructed all the SEEG electrodes in our cohort from post-CT imaging in about 1s per patient. Moreover, by coregistering post-implant CT data to pre-implant MRI scans, we were able to accurately map each channel position to its relative anatomical region (Figure 2(a)), which can be easily derived from pial surface mesh with Freesurfer suite (http://freesurfer.net/). Thus, the proposed method will help the interpretation of the acquired local field potentials by directly and accurately linking the acquired signals to their neural sources which itself improve the localization process.

Furthermore, the DEETO software outputs the estimated position a text file containing for each contact its label and its coordinates in RAS space within single subject CT/MRI reference space. The current implementation also provides either a single mesh file for each electrode or a single mesh file for the whole implant as VTK polydata file. These data represent the contacts with their real geometry (refer to methods for details) and the coordinates, in both text and vtk files, are relative to CT original geometrical space.

### Error against manually segmented points

We tested the error of the implemented algorithm against manually localized contacts. A pool of trained neurosurgeons have manually segmented 3 to 4 multilead electrodes from subgroup of 12 subjects (total subjects 28), 503 contacts overall (total contacts 5937). The manual segmentation process took 1h per subject resulting in a total of approximately 28 hours of two skilled neurosurgeons while the implemented tool required only 0.8s per subject to perform the same task. All the true contacts have been successfully identified with a localization error of 0.5 mm ± 0.06 mm (mean ± s.d.). We do not see the need for a manual post-processing correction for errors of this magnitude - both because the error is so small and because it is unclear whether the error is actually attributable to the algorithm or to the manually segmented points. In clinical use, this error is irrelevant because the resected region is much larger, in the scale of several centimeters, than the localization uncertainty of any single contact.

### Robustness to target-point displacements

The proposed algorithm depends on the target-entry axis to initialize the search space.

The information already available at trajectory planning stage can be used as reliable entry point estimate, since its position is generally more accurate (*i.e.*, screw attached to the skull) than it is for target points. In fact, planned target points can sensibly deviate from planned positions, with displacements of several millimeters up to tens of millimeters [13].

We created 40 new fiducial list files for each of the 28 subjects with 8 target point displacements ranging from 1 to 15 mm and 5 points each. Each new target point has been chosen randomly on the target-point-centered circle of fixed radius (*i.e.*, 1 to 15 mm) laying on the perpendicular plane to the target-entry axis.

We quantified the error as a function of target point displacements, as well as the false positive (FP) and false negative (FN) rates. These last were quantified as the number of FP and FN out of the total number of channels for each subjects. We reported in Figure 2(b) the average across subjects along as 95% and 5% confidence bounds. We report that the error of the algorithm is above 0.005 mm for displacements up to 12 mm, and less than 0.02 mm for displacements up to 15 mm. Moreover, the FN and FP are less than 10% and 7% for target displacements of 15 mm (Figure 2(b)). These are mostly due to the fact that initial entry and target points are used as initial estimation of real electrode axis, whose direction erroneously falls in the background causing the algorithm to stop. A more complex initial estimation process could be used to sensibly reduce the FN and FP rate.

It has to be noted that, the above reported algorithm performances have been computed using information that were available at trajectory planning stage where target end entry points could actually be different from their final positions. The inclusion of information on post-implant localization of entry and target points would strongly increase the algorithm accuracy.

Moreover, with the current results on algorithm robustness we believe that even in presence of missing contact the amount of time saved would sensibly benefit the clinical routines. It further have to be noted that FN and FP rates are computed considering that all electrode positions have been displaced of that amount. This case is unrealistic but serves our purpose to show the robustness of our approach. In normal cases, at most one electrode undergoes such large deviation from planned trajectory. Thus, we feel that the number of missed contacts in a more realistic case would indeed not cause problems to the subsequent analysis.

### Algorithm reliability to specific domain problems

In implantation of multiple SEEG electrode shafts, contacts belonging to different electrodes can be so close that they seem to touch each other. These effects required particular attention and specific designs in order to be overcome. In general, the vast majority of SEEG electrodes fulfill the generic assumptions that (a) the axis of an electrode cannot be a curve; (b) two electrodes cannot cross each other, and (c) each electrode must have a unique axis.

**Figure 3:**
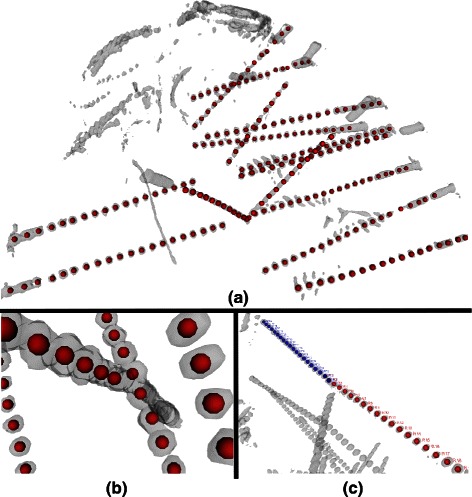
**An Example of the result of algorithm reconstruction.** The reconstructed centroids (red or blue spheres) are confidently representing the center of each original contact (shaded gray) visually assessing the accuracy of the method **(a)** even in presence of SEEG specific problems such as **(b)** crossing or **(c)** touching electrodes.

However, the presented method even in presence of curved electrodes correctly reconstructs the electrode axis and each contact (Figure 3) by redefining at each step the electrode axis which more closely resemble the real geometry.

During the implantation of multiple electrode shafts, it is common that several electrodes have crossing and/or nearby trajectories. This effect combined with metal artifact results on “merging contacts” that become a single mass of voxels. Our SEEG customization correctly handles these situations by iteratively refine the centroid computation in the presence of displacements between adjacent contacts that result on angles greater than 10 degrees (Figure 3(b)).

While only in rare cases, a patient can been implanted in both hemispheres. Therefore, it indeed might occur that electrodes coming from the opposite sides seem to lay on the same axis. Moreover, in these cases the two tips affected by metal artifacts are so narrow that the ensemble of all the contacts seem to belong to the same long electrode traversing the whole brain volume. Thus, difficulties arise trying to automatically discern between the two electrode tails.

In our cohort, we did not find any electrode shaft implanted in one hemisphere to have contacts in the other hemisphere. Thus, we fixed the mid-planes dividing the two hemisphere as end-point of each iteration. Under this assumption, our algorithm performs correctly even in presence of several electrodes with the same axis (Figure 3(c)).

## Conclusions

The manual neuroanatomical localization of SEEG contacts is a challenging, tedious and error-prone task that consumes several hours of working time of highly trained clinicians. At present, there are no robust tools or techniques to support clinicians in this task.

We presented here an automated segmentation algorithm specifically designed to localize SEEG contacts from a thresholded post-implant Cone-Beam CT volume. The proposed method reliably identifies SEEG contacts and co-localizes them with MRI-derived volume and surface segmentations of cerebral structures with sub-millimeter accuracy, which opens new avenues for exploiting the anatomical accuracy of SEEG in both clinical use and neuroscience research. The presented method is robust to electrode deviations up to 15 mm from the pre-surgically planned trajectory. Finally, the method is automatized so that non-experts such as students or technicians can easily and reliably carry out the segmentation task.

The proposed method hence at a greatly decreased human resources cost yields significantly improved neuroanatomical localization accuracy for SEEG electrode contacts.

## Method

This section is organized as follow: (i) we first give some basic notations that will be used; (ii) then we present the algorithm for the automatic contact segmentation, along with a detailed description of SEEG-specific design, and finally (iii) we present DEETO, *i.e.*, the prototype that implement the algorithm. The study was approved by the ethical committee of Niguarda Hospital “Ca’ Granda”, Milan, and was performed according to the Declaration of Helsinki. All the patients participating to the present study have given written informed consent for the use of their data for research purpose.

### Basic notations

#### Data acquisition and subject details

In the present study we analyzed data from 28 subjects of which 13 male and 15 female of 28.89 year in average ranging from 17 to 47 years. The full details about SEEG implant approach and all the required steps that lead to a successful implant can be found elsewhere (see [13]). Here we briefly give some details to clarify the context.

During planning of SEEG implants, neurosurgeons define a set of trajectories, one for each electrode, in order (i) to reach the pre-defined targets (ii) avoiding visible arteries and veins. These trajectories are then provided to a robotic stereotactic system (Neuromate, Renishaw mayfield), able to align its tool holder along the vector of the planned trajectory. The vector is defined as two points, the entry and the target points, which are the most proximal and most distal points with respect to cortical surfaces, respectively.

After surgical implant, the electrode positions are visually assessed on a CT scan acquired directly in the operating room (OR). Moreover, the planned entry and target points are then saved on a ASCII text file for offline processing in the presented algorithm as initial estimation of electrode axis.

Thus, in our framework we extract contact coordinates fusing the two CT volumes acquired in the OR before (pre-CT) and after (post-CT) surgical implantation of intracerebral electrodes. Pre-CT is routinely acquired to register the patient’s head to the surgical reference space [13].

After a preliminary registration (Correlation Ratio, 6 Degrees of Freedom (DOF)), we subtract the pre- from the post-implant CT scan in order to delete the bone tissue since its voxels have intensities in the same range as the contacts.

Furthermore, to increase the contrast between contacts and brain tissues, we threshold the images with a single threshold filter using 1600 as cut-off value (Figure 1(a)). The voxels laying above the threshold maintained their original value, while the voxels below the threshold have been set to 0.

The fiducial file containing the planned entry and target points is created with 3DSlicer (http://www.slicer.org) and contains two types of information (1) comments (each line starting with #), and (2) several pairs of points with the following format:

$$label,x,y,z $$

where label represents the unique electrode name and (*x*,*y*,*z*) the coordinates in millimeters of either the target or entry point.

In the presented study we consider electrode shafts containing 5–18 contacts (DIXI Medical, Lyon) where each contact is a platinum-iridium cylinder of 0.8 mm diameter, 2 mm long with 1.5 mm of inter-contact distance. Conventionally, we mark each electrode with a capital letter and each contact belonging to the electrode with an increasing integer from 1 to *N* depending on electrode model. We define as the first contact (*i.e.*, 1) the one on the tip of the electrode shaft, while the last contact (*i.e.*, *N*) is the most superficial one closer to the screw.

#### Definitions

We consider an image as a set of points, which can be described by a tuple

(1) $$ (x, y, z, I(x,y,z))  $$

where (*x*,*y*,*z*) are the coordinates of a point in the euclidean *R* ^3^ space, while *I*(*x*,*y*,*z*) is the intensity function over the same space. Given a point *p*(*x*,*y*,*z*), I(p)(or I(x,y,z)) represents the intensity value in *p*. For sake of simplicity in the following we can refer to an image with its intensity function. Two points, *p*_1_(*x*_1_,*y*_1_,*z*_1_) and *p*_2_(*x*_2_,*y*_2_,*z*_2_), define a straight line having the following parametric representation:

(2) $$ \begin{array}{c}\begin{array}{c}p={p}_1+{\overline{v}}_{p_2,{p}_1}\cdotp t={p}_1+\frac{p_2-{p}_1}{\parallel {p}_2-{p}_1\parallel}\cdotp t\\ {}\end{array}\end{array} $$

The point *p* with distance *d* from *p*_1_ along $v_{p_{2},p_{1}}$ can be computed by the following equation:

(3) $$ p = p_{1} + \overline{v}_{1,2} \cdot d   $$

Given the intensity function *I*(*x*,*y*,*z*), we define the moment of order *p*+*q*+*r* in the equation:

(4) $$ M_{pqr} = \sum\limits_{x} \sum\limits_{y} \sum\limits_{z} \! x^{p} y^{q} z^{r} I(x, y, z) \,  $$

The moment of order 0, *M*_000_ of a given 3D image is defined as

(5) $$ M_{000} = \sum\limits_{x} \sum\limits_{y} \sum\limits_{z} \! I(x, y, z) \,  $$

and it represent the total mass of an image having intensity function *I*(*x*,*y*,*z*). There are three different moments of order 1 in a given 3D image, and they are defined as:

(6) $$ \begin{aligned} M_{100} &= \sum\limits_{x} \sum\limits_{y} \sum\limits_{z} \! x \cdot I(x, y, z) \\ M_{010} &= \sum\limits_{x} \sum\limits_{y} \sum\limits_{z} \! y \cdot I(x, y, z) \\ M_{001} &= \sum\limits_{x} \sum\limits_{y} \sum\limits_{z} \! z \cdot I(x, y, z) \\ \end{aligned}  $$

A region *R* of an image *I* in *R* ^3^, can be defined as a subset of points of the image. A cubic region *R* of an image *I*, centered in *c*, and with a side length *l*, *R*(*c*,*l*), is a set of the points of the image belonging to the cube. The center of mass of a three dimensional region *R* is defined as

(7) $$ {c^{R}_{m}} = \left(\frac{M^{R}_{100}}{M^{R}_{000}},\frac{M^{R}_{010}}{M^{R}_{000}},\frac{M^{R}_{001}}{M^{R}_{000}}\right)  $$

where each moment is computed with respect to the region *R*.

We define the threshold**S** as the value in the range of the intensity function, that marks the line between points belonging to an electrode (intensity value above S) from points belonging to the background. In our dataset S is always 1600, *i.e.*, the threshold value used to filter the CT images before the segmentation. However, to be more general, in our framework we pick as threshold **S** the first local minima in the image histogram.

### Automatic segmentation

#### Algorithm overview

**Figure 4:**
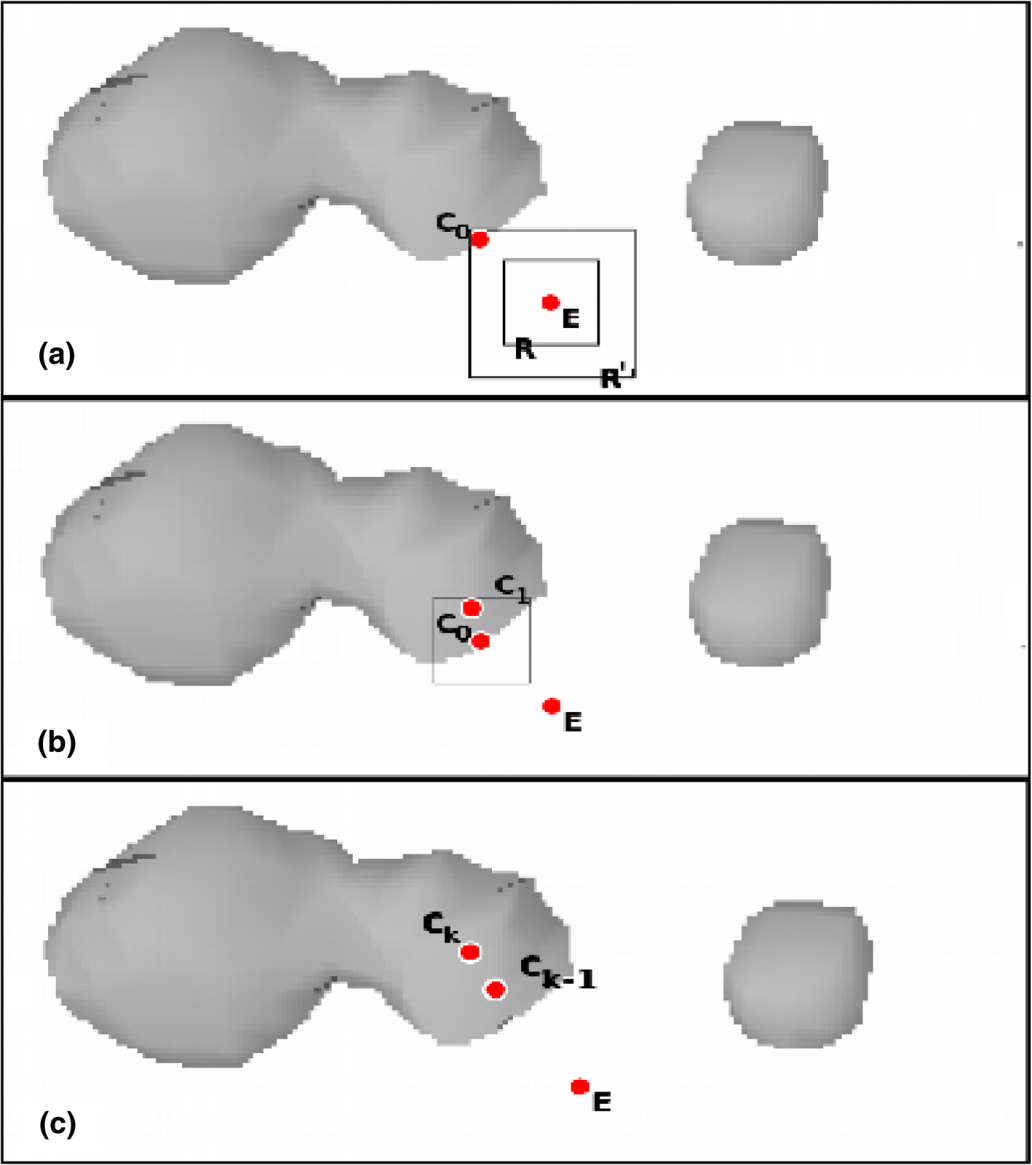
**An example of the head point computation of the Electrode Axis Estimation step.** **(a)** in a region *R* the algorithm looks for a point, *C*_0_, with an intensity value greater than the threshold. If such point does not exist the region size is increased, *R* ^′^, until the point is found. In **(b)** the algorithm compute the center of mass of a region *R* centered in *C*_0_. This step is iterated, *C*_*k*_, until the point *C*_*k*_ is equivalent to *C*_*k*−1_. In **(c)** the point *C*_*k*_ will be used as *head point* point (*H*) in later analysis steps.

**Figure 5:**
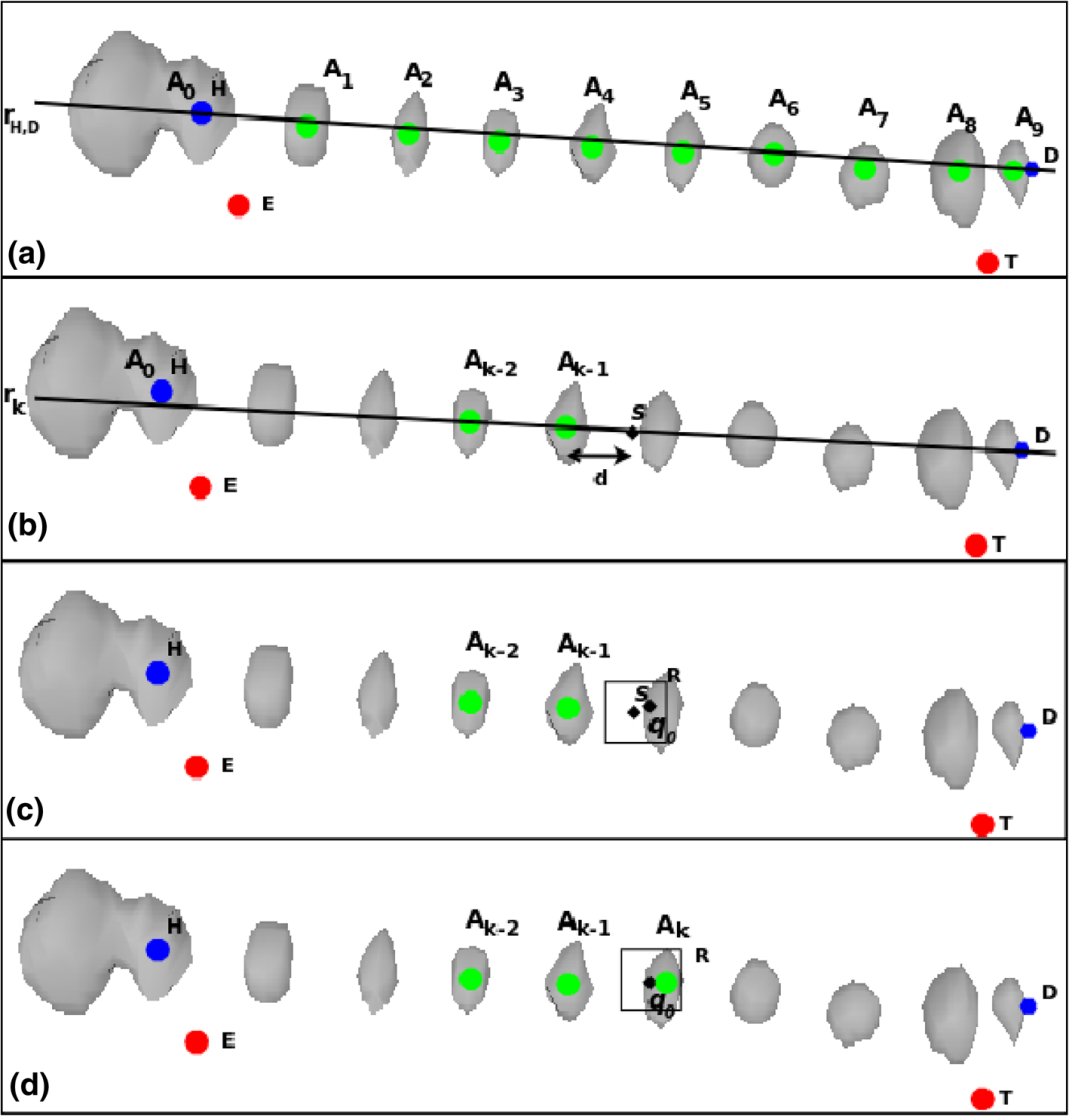
**An example of the behaviour of the algorithm in the Electrode Axis Estimation step.** **(a)**This panel represents the result of the algorithm, *i.e.*, the axis (*r*_*H*,*D*_) and each axis point, *A*_*k*_, computed at each iteration, while the other three figures represent the steps executed at each iteration: **(b)** represent the computation of a point *s*, on the line connecting the two previous computed axis points, *A*_*k*−1_ and *A*_*k*−2_, with distance *d* from the point *A*_*k*−1_; **(c)** is the computation of a point *q*_0_ that it is the point with the higher intensity value in “cubic” region *R* centered in *s*; **(d)** represent the computation of the axis point *A*_*k*_, *i.e.*, the center of mass of a region *R* centered in *q*_0_.



The goal of the algorithm is to segment SEEG electrodes from post-implant CT scan, as the one presented in Figure 1(a). In this section we present the algorithm which initially assumes that: (a) the axis of an electrode cannot be a curve; (b) two electrodes cannot cross each other, and (c) each electrode must have a unique axis. For each electrode the algorithm executes two main steps: electrode axis estimation (step 1, S1) and electrode contacts segmentation (step 2, S2). The axis estimation needs two points: the *head point* and the *tail point* which we define as the most superficial and the deepest points belonging to the electrode, respectively. Thus, the electrode axis estimation is further divided in two sub steps: (S1.1) the estimation of the *head point*, and (S1.2) the estimation of *tail point*. These two sub steps are implemented in the functions LOOK4HEAD and LOOK4TAIL, respectively, and are presented in Figures 4 and 5. In the second step, electrode contacts segmentation, the algorithm iteratively estimates the position of each contact within a geometrical-constrained search space by means of the NEXT function. The search-space is defined by two strong constraints. The former represents the fixed inter-contact distance (*i.e.*, the distance between two subsequent contacts); the latter states that the axis deviations which can only occur within electrode cables connecting two adjacent contacts, should be minimal (*i.e.*, <10 deg). This geometrical-constrained estimation of each electrode contacts is handled in ELECTRODECONTACTSEGMENTATION function (see Algorithm 1 from line 6 till line 13).

#### Electrode axis estimation: head point computation - S1.1

In most cases both the *planned entry point* and the last (*i.e.*, the most superficial) recording contact are masked by metal artifacts surrounding the screw used to fix the electrode to the skull. Nonetheless, in order to estimate the head point position is sufficient to find approximately the center of the screw. We proceed by searching for a cubic region with the highest intensity nearby the *planned entry point* and using its center of mass as first approximation as can be seen in Figure 4. To this aim, starting from the *planned entry point*(*E* in Figure 4(a)), we search for a voxel p with significant (*i.e.*, I(p) >S) value within a cubic region (*R*) of 3 mm centered in *E*. If no points within the region exceed the threshold, we enlarge with 1 mm step the region length up to 10 mm, *R* ^′^, until a point is found (Figure 4(b)). From this initial candidate, *C*_0_, we iteratively compute the center of mass *C*_*k*_ of a region *R* centered in *C*_*k*−1_ using the equation in 7 until the contact centroid *C*_*k*_ is equal to *C*_*k*−1_±*ε* (Figure 4(c)). This final point, *C*_*k*_, is the approximated head point (*H*) position.

#### Electrode axis estimation: tail point computation - S1.2

While the *planned entry point* displacement is a rare event, since it can only deviate from its original position due to surgical reasons, the displacement of a *planned target point* indeed might occur more often. However, for what concerns the *tail point* estimation, the approach adopted for the *head point* estimation has a major drawback in case of several converging electrodes. This way, looking for the center of mass of the region with the highest intensity in the neighboring of the *planned target point* might results on unrealistically deviated contacts which affects the whole search.

This drawback is indeed much more evident in SEEG implants compared to other iEEG modalities where several electrodes are routinely used to target the same region from different sides and rarely even from opposite hemispheres. In order to estimate the tip of the electrode, D, the algorithm uses the *H* position and the estimate *r*_1_, represented by the line connecting *H* and the planned target point *T*. The algorithm iteratively try to follow the electrode shape individuating subsequent point, *A*_*k*_, belonging to the electrode until the tip *D* is reached, see Figure 5. Each point *A*_*k*_ is computed by executing the following steps:

- A point *s*_*k*_ with distance *d* from *A*_*k*−1_ is computed, laying on the line *r*_*k*−1_ connecting two subsequent points, *A*_*k*−1_ and *A*_*k*−2_. The algorithm starts with *A*_*o*_=*H*, and *r*_0_ represented by the line connecting *A*_0_ and *T*, the planned target point.
- Starting from a generic *s*_*k*_, as in the *H* computation phase *S*1.1, a cubic region (*R*) centered in *s*_*k*_ is defined and iteratively enlarged until a significant voxel *q*_0_ is found (*I*(*q*_0_)>*S*). A graphic representation of this step is in Figure 5(c).
- Starting from *q*_0_, the algorithm iteratively compute the center of mass *q*_*j*_ of a region *R* centered in *q*_*j*−1_ using Equation 7, until the difference between two subsequent centroids is less than a threshold *ε*, *i.e.*, ∥*q*_*j*_−*q*_*j*−1_∥<*ε*. The final centroid *q*_*j*_ represents the position of the new point *A*_*k*_. A graphic representation of this step can be found in Figure 5(d).

#### Electrode contacts segmentation - S2

Once the position *D* of the tip of the electrode has been estimated, it is possible to start the process of contact segmentation. The algorithm is the same adopted in phase S1.2 for tail point computation. In this case the starting point *C*_0_ is the tip *D* and the starting search direction is from *C*_0_ to *H*. The only exception is in the computation of the first point *s*_0_: since *D* represents both the tip of the electrode and the tip of the contact, *s*_0_ has distance $\frac {d}{2}$ from *C*_0_, where *d* is the distance between two subsequent center of contacts.

### SEEG-specific methods

In describing the algorithm we have not made any assumptions about the electrodes, except the possible deviation of the target points from their planned positions. However, post-CT scans are affected by three major problems: (a) the axis of an electrode could be a curve, (b) two electrodes can apparently cross each other, (c) two electrodes can lay on the same axis.

#### Curved electrode

Curved electrode may arise when during surgical implant the tip of the electrode encounter hard tissues or simply end up in a sulcus. If the algorithm deals with a fixed electrode axis it may fail to find a solution since the region *R* may not contain points above the threshold and thus the resulting center of mass will be null. In these cases the algorithm stops even if the contacts are not yet all discovered. In our algorithm the axis is represented as piece-wise linear function, where each segment is the conjunction of two subsequent contacts, *C*_*k*−1_, and *C*_*k*−2_. This allows to follow precisely the curvature of the electrode.

#### Crossing electrodes

When an electrode cross another electrode, the crossing contacts are merged and they became an indistinguishable big contact (Figure 1(b)). In this case the center of mass of a region *R* constructed around this big contact may produce a wrong center of mass and thus a wrong contact. This problem may impair the computation of the next contact points, resulting in errors in the reconstruction. However, given the fixed geometry of an electrode, a contact *C*_*k*_ cannot be too far from the line passing for the two previous points, *C*_*k*−1_ and *C*_*k*−2_, *i.e.*, it cannot deviate too much from its expected trajectory. Indeed we experimentally see that given a contact *C*_*k*_ the angle between the two lines $r_{C_{k},C_{k-1}}$ and $r_{C_{k-1},C_{k-2}}$ is at most 10 degrees. If the angle is greater than 10 degrees it means that a contact is crossing another contact and we must reduce the amount of points in *R*, or in other words, we have to reduce the size of *R*. More in details we iterate the lines 8 and 9 of the algorithm (see Algorithm 1) by decreasing the size of *R* each time, until either the angle between the lines $r_{C_{k},C_{k-1}}$ and $r_{C_{k-1},C_{k-2}}$ is less than 10 degree, or *R* has size 0.

#### Electrodes on the same approximate axis

This problem can be seen in Figure 1(c) and typically is posed by two electrodes having approximately the same axis, which happens, *e.g.,* when the target point is in deep structures and is approached bilaterally. In this case two different electrodes seems merged into one long electrode since their tip is very close to, if not crossing, each other. However in case of reliable *planned target point* this would not be a problem, but the *tail point* computation fails because the step S1.2 cannot distinguish one electrode from the other, and cannot correctly stops. In order to solve this problem we assume that an electrode cannot pass the medial longitudinal fissure, *i.e.*, cannot pass from one hemisphere to the other. Thus we modify the stop criteria of the *tail point* computation, step S1.2, by adding the condition that an electrode cannot be into two different hemispheres at the same time. To do this we add the computation of the geometrical plane to which belong the medial longitudinal fissure, and then, given a contact, we compute the difference between the distance of this plane from the *head point*, and the distance of the contact from the *head point*: if it is negative than the contact pass the hemisphere and the search must stop, otherwise it can continue.

### DEETO

seeg electroDE rEconstruction TOol (DEETO) is an open-source software that implement the advanced algorithm presented above. It is written in C++ on the top of some libraries presented below. It takes the following inputs:

- A 3D image, *i.e.*, a pre-filtered post-implant Cone-beam CT scan.
- A fiducial file where are stored the name and the coordinates of the *planned entry point* and *planned target point* for each electrode. By default, the coordinates are suppose to be in CT space.
- A bunch of switch, that are self explained in the readme file. The most used one is the flag -r (–noref) used to indicate that in the fiducial file the points coordinates are not in the CT space.

DEETO output can be of two types:

- A simple txt file where each contacts is stored and written using the same format, and the same coordinate system, of the fiducial file; or/and
- A single vtk polydata file that represents the triangular meshes of all the electrodes in their estimated positions or a separate vtk polydata file for each electrode.

#### Download

DEETO source code can be freely downloaded from the following web-page: https://github.com/mnarizzano/DEETO.

#### Dependencies

For building DEETO is necessary to install the following libraries and/or tools:

- CMake, version 2.8 downloadable from http://www.cmake.org/cmake/resources/software.html. CMake is an extensible, open-source system that manages the build process in an operating system and in a compiler-independent manner.
- Insight Toolkit (ITK) version 4.3.1 downloadable from http://www.itk.org/ITK/resources/software.html. Is an open-source software toolkit for performing registration and segmentation of a digitally sampled representation.
- tclap, Templatized C++ Command Line Parser, version 1.2, downloadable from http://sourceforge.net/projects/tclap/files/, is a simple templatized C++ library for parsing command line arguments.
- Visualization Toolkit library (VTK), version 5.6, downloadable from http://www.vtk.org/VTK/resources/software.html. It is an open source, freely available software system for 3D computer graphics, modeling, image processing, volume rendering, scientific visualization and information visualization.

#### Build and run

Build the project with cmake by running the following commands

cmake CMakeLists.txt

makeand finally run the generated executable file in directory bin by using the following command line: deeto -o <file out name > -f <fiducial file input name > -c <CT image in name >.
